# Challenges of brain-computer interface facilitated cognitive assessment for children with cerebral palsy

**DOI:** 10.3389/fnhum.2022.977042

**Published:** 2022-09-20

**Authors:** Jane E. Huggins, Petra Karlsson, Seth A. Warschausky

**Affiliations:** ^1^Direct Brain Interface Laboratory, Department of Physical Medicine and Rehabilitation, Neuroscience Graduate Program, University of Michigan, Ann Arbor, MI, United States; ^2^Direct Brain Interface Laboratory, Department of Biomedical Engineering, Neuroscience Graduate Program, University of Michigan, Ann Arbor, MI, United States; ^3^Theme Technology, Faculty of Medicine and Health, Cerebral Palsy Alliance, The University of Sydney, Sydney, NSW, Australia; ^4^Adaptive Cognitive Assessment Laboratory, Department of Physical Medicine and Rehabilitation, University of Michigan, Ann Arbor, MI, United States

**Keywords:** assistive technology, neuropsychology, disability, event-related potential, pediatric, choice-making, attention, P300

## Abstract

Brain-computer interfaces (BCIs) have been successfully used by adults, but little information is available on BCI use by children, especially children with severe multiple impairments who may need technology to facilitate communication. Here we discuss the challenges of using non-invasive BCI with children, especially children who do not have another established method of communication with unfamiliar partners. Strategies to manage these challenges require consideration of multiple factors related to accessibility, cognition, and participation. These factors include decisions regarding where (home, clinic, or lab) participation will take place, the number of sessions involved, and the degree of participation necessary for success. A strategic approach to addressing the unique challenges inherent in BCI use by children with disabilities will increase the potential for successful BCI calibration and adoption of BCI as a valuable access method for children with the most significant impairments in movement and communication.

## Introduction

**FIGURE 1:**
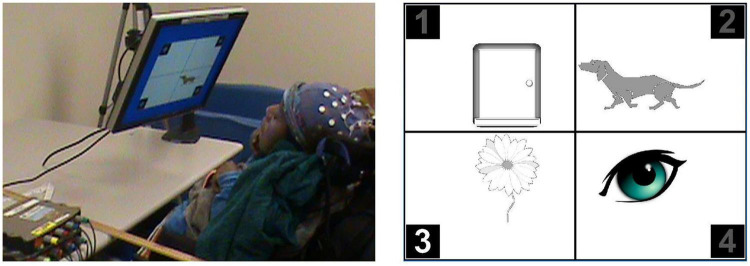
Left: Participant with cerebral palsy (CP) viewing first calibration screen. Adjustable monitor support enables optimal positioning. Right: Closeup of a later calibration screen, with the stimulus flashing for picture 3.

Brain-computer interfaces (BCIs) have long been considered communication tools for people with impairments that prevent verbal communication and manual computer access. The event-related potential BCI design introduced by Farwell and Donchin (1988) as the P300 BCI design has been used for daily communication in home environments by people with amyotrophic lateral sclerosis (ALS) (Sellers et al., 2010; Wolpaw et al., 2018). This non-invasive BCI design (Figure 1, left) presents stimuli on a computer screen, senses brain activity using electroencephalogram (EEG) electrodes, and interprets the EEG to determine to which single stimulus the user is paying attention. Although BCI has been successful for able-bodied adults and adults with acquired impairments, little is known about BCI use by children with severe multiple impairments who need technology for communication (Orlandi et al., 2021).

Cerebral palsy (CP) is one of the most common childhood disabilities (Odding et al., 2006). Approximately 25% of people with CP cannot talk (Novak et al., 2012; Acpr Group, 2013), while 15–19% have no method of communication (Parkes et al., 2010). With verbal comprehension often exceeding expressive capacities in children with complex communication needs (Sevcik, 2006), we created and studied (with publisher’s permission) BCI-facilitated access (Alcaide-Aguirre et al., 2017) to a standard receptive vocabulary test, the Peabody Picture Vocabulary Test, 4th edition, PPVT-4; (Dunn and Dunn, 2007). EEG was recorded with 32-location gel-based EEG caps (Electro-cap International, Inc., Eaton, OH, United States) and g.USBamps (g.tec medical engineering GmbH, Schiedlberg, Austria). Directing attention among four images in response to a recorded verbal prompt (Figure 1) enables BCI-facilitated access (Huggins et al., 2015). We used this access method with children and young adults (Alcaide-Aguirre et al., 2017), both typically developing and with CP, who could also complete the standard test format as a necessary first step in validating the new access method (American Educational Research Association et al., 1999). The 29 recruited participants, 18 with CP, were age 8–27 years (mean 15.0, SD 5.5). Primary or secondary CP type included spasticity in 88.9%, with primary or secondary ataxia in 11.2% and 5.6% dystonic. Primary or secondary tone was hemiplegic or bilateral spasticity, with 66.7% exhibiting hemiplegia and 33.4% exhibiting diplegia or quadriplegia. Functional mobility levels using Gross Motor Functional Classification System (GMFCS) (Palisano et al., 1997) scores were: level I-II 76.2%, level III 14.3%, and level IV-V 9.6%. Functional dexterity ratings using the Manual Ability Classification System (MACS) (Eliasson et al., 2006) included level I 71.4, level II 19.0, and level III 9.6%. The study focused on the psychometrics of BCI access to the PPVT-4, showing excellent measurement agreement with standardized administration (Warschausky et al., 2022). Our next step was evaluating BCI-facilitated tests with those for whom standardized test administration was inaccessible (Huggins et al., 2020). The 28 recruited participants were 10–43 years (mean 19.5, SD 9.4) with GMFCS scores of Level IV 17.9% and Level V 82.1% and MACS scores Level IV 28.6% and Level V 71.4%. Primary tone was spasticity (81.5%) and dystonia (29.6%) with 76.2% exhibiting quadriplegia, 19.0% diplegia, and 4.8% hemiplegia. A separate effort, involving 18 participants to date (age 13–79 years; mean 40.7 ± 18.6), is examining BCI access to a commercial speech generating device (Huggins et al., 2019) with 7-or 16-location gel electrodes or seven-location dry electrodes (VR300 amplifier from Wearable Sensing, San Diego, CA, United States). Here we discuss the challenges experienced with the 75 participants in these studies.

## Challenges and compensatory strategies

### Caregiver participation

The participation of trusted adults is important for any BCI use by children, especially children with multiple impairments. In our BCI lab, parents/guardians receive advance information about the BCI during the consent process, which may help set expectations. Parents have identified topics such as football, pictures of food, or specific cartoon characters as content that is motivating or of interest to a child to establish rapport and reduce potential participation anxiety. It is recommended that future studies systematically gather this type of information and incorporate the content into initial introductory materials and training stimuli. In the session, caregivers familiar with the child help to manage expectations regarding BCI procedures, assist with interpreting non-verbal communication, provide comfort, entertain the child during BCI setup, or focus their attention on a task. However, caregivers also can be a distraction or provide inaccurate instructions by well-meaning verbal or physical prompts.

### Situational anxiety and motivation

Despite caregiver presence, some children still exhibited signs of anxiety when entering the study site. Children often need advance preparation for unfamiliar settings and task demands, which could be accomplished with video orientation materials (see Section “Comprehension of task”) and guided parent task-specific behavioral modeling. We have found that taking the BCI to the child’s home or other familiar environment can help alleviate anxiety. Children’s assent for participation should always be sought, although children without well-established communication methods will likely be unable to pose questions and only express lack of assent or a desire to end participation with non-verbal behaviors. These may include closed eyes, turning away from the screen or experimenter, or pretending to sleep.

Children’s willingness to use assistive technology also depends on their perception and experience of immediate benefits (Hemmingsson et al., 2009). In addition, there can be self-image related resistance to using unusual technologies in social situations (Hemmingsson et al., 2009; Murchland and Parkyn, 2010). Furthermore, there are individual differences in mastery motivation, or the willingness to persist in the face of the challenge of learning new technology.

### Task demands

All elements of a BCI task should be evaluated for familiarity and fit with capabilities and experience. Before working with children who could not access the standardized vocabulary test, we carefully selected calibration words the children were likely to have experienced in either personal, educational, or entertainment contexts. Starting from word frequency lists, we excluded items that involved motor demands inaccessible to the child or with associated images that were ambiguous or scary. For this cross-cultural study, we also removed words with inconsistent meanings between the United States and Australia (e.g., cookie vs. biscuit). Illustrations were selected instead of photographs to facilitate the planned transition to the PPVT-4 vocabulary test (Dunn and Dunn, 2007), which uses illustrations.

A child who has never successfully used technology should not be expected to be immediately ready to follow multi-step instructions for BCI operation. However, gradual introduction of concepts involved in BCI use may be possible. During calibration, we supported the concept of picture identification by using color to highlight the target among grayscale non-targets. Further, we started with a single picture and three empty quadrants (Figure 1, left), then a picture with non-target generic shapes, and finally a target picture with three non-target pictures (Figure 1, right). Similarly, we first used a + character on the flashing labels before introducing the numeric labels that matched the PPVT-4 testing screens. However, the efficacy of this graded training is not yet clear.

### Experience with choice-making

The most fundamental skill for participation in cognitive assessment or BCI use is the ability to make a choice (Van Tubbergen et al., 2008). In typically developing children, choice-making skill follows a developmental progression, from basic orienting, to preference, to directed choice in response to questions requiring indirect application of knowledge (e.g., can dogs fly?). Directed choice-making is needed for typical cognitive assessments. Yet, it can be difficult to determine if children with limited reliable overt communication have achieved directed choice-making. This is particularly true for children with CP who function at GMFCS (Palisano et al., 1997), MACS (Eliasson et al., 2006), and Communication Function Classification System (Hidecker et al., 2011) levels IV-V, which indicate inability to reliably move or talk without assistance (if at all). A child skilled with a communication device exhibits directed choice-making. However, children with lower levels of motor and communication function in effect can be “locked in” with limited recognition of their underlying capabilities. Further, without choice-making opportunities, they can be completely naïve to the most fundamental testing demands. Thus, even the initial preparatory and learning/practice trials may provide novel information regarding choice-making capabilities.

### Screen layout and brain-computer interface stimuli

In our cognitive testing BCI design, the spatial separation between the vocabulary pictures and flashing labels (stimuli) added to the complexity of instructions. We subsequently considered (but have not yet tested) alternate stimulus types and locations. Face stimuli have been shown to improve BCI accuracy, especially for people with disabilities (Kaufmann et al., 2013). However, face stimuli may increase the complexity of instructions. We prefer the idea of integrating stimuli into the pictures or their backgrounds or using actual or simulated motion as the stimulus (Martens et al., 2009; Liu et al., 2010). If the stimulus is a small picture rotation, the user could be simply instructed to pay attention to the picture that matched the word and the response to the “movement” of the picture should be largely automatic.

### Comprehension of task

To generate P300’s, the user must select a specific stimulus among the possible stimuli, monitor for occurrences of that stimulus, and preferentially attend to that stimulus. This process is often explained by instructions to count the flashes of the specific stimulus. The typical practice of referring to stimuli as flashes seems readily understood by children. However, parents report that children may not be able to count or may find counting stressful because of association with the demands of school. Further, children who are able may prefer to count out loud, which could generate EEG artifact. Yet, instructions other than counting the flashes are not as easily understood. Several variants have produced inadvertent, non-useful results. Participants instructed to think “yes” when a flash happened in the right place reported that they were thinking yes over and over again, but their EEG showed a lack of stimulus-related responses. Another participant responded to the stimulus, but the response was evident because the participant performed the physical movement used to indicate yes, a source of both fatigue and artifact. Another participant who was non-verbal made sounds in response to the stimulus. Several of these participants generated clear responses when asked to try counting instead. We have also tried the instruction to think “now” when a flash happens in the right place, this has the advantage of describing the connection between stimulus and mental response. Sometimes, thinking the name of the target item when the target stimulus occurs is sufficient, although this instruction may easily be misunderstood as thinking the name of the item repeatedly (without the vital connection to the time of the stimulus).

A key difficulty is the impossibility of demonstrating P300 BCI use. Most instructions for operating devices, even assistive technology, involve some task-specific behavioral modeling so the learner can mimic the actions required for use. But anything added to BCI use to indicate the thought process involved artificially inserts a physical response that is unnecessary and potentially problematic, both for a user with a disability and for EEG quality. To address this, we are creating instructional videos for each BCI study that include slow motion sections emphasizing the relationship between stimulus timing and mental response. Videos also enable the use of thought bubbles or other representations of thought to model mental responses to target stimuli e.g., (Branco et al., 2021).

### Age and attention

Even children who try to follow instructions may struggle with doing so long enough to provide 10–20 min of calibration data. Our study found that children in the 8–10-year age range exhibited significant inattentiveness during calibration. Children could sometimes be redirected toward the calibration task by prompting. Also, breaking the calibration data into shorter segments was sometimes of benefit. However, inattentiveness and/or reported lack of interest were an ongoing barrier.

Immediate feedback from the system on whether they are doing something wrong and how to change it would be desirable (Taherian and Davies, 2018). An example of the effect on mental tasks was demonstrated (Schudlo and Chau, 2014) where eight out of 10 participants adjusted their mental strategies when receiving feedback. However, prior to BCI calibration, relatively little information is available to inform specific feedback.

### Processing speed

Individual differences in cognitive processing speed can affect BCI accessibility. Children with CP are at significant risk for slowed processing speed, even with normal range IQs (Kaufman et al., 2014). Processing speed can affect both the time to register a stimulus and the time to plan the next target after the BCI registers a selection. The duration of BCI stimuli can be adjusted to accommodate slower visual processing speed. However, appropriate planning time is an often overlooked and potentially more complex challenge. P300 BCIs usually operate in a synchronous mode in which a specific number of stimuli are presented, a decision is made, and stimuli for the next selection start after a fixed duration pause. Two approaches might accommodate an individual’s slower processing speed. The time between selections can be manually adjusted. Alternatively, automatic algorithms might identify whether an EEG response was generated to one of the stimuli. Our cognitive testing BCI used statistical analysis of the stimulus responses to identify when one stimulus produced a significantly larger EEG response (Alcaide-Aguirre et al., 2017; Aref, 2018). This enabled the user to spend as much time as needed to consider the illustrations before picking the one they wanted to select and attending to the associated stimulus.

### Calibration time

An advantage of the P300 BCI design for children is that it can be calibrated within a single session as compared to the multiple sessions generally required for motor imagery BCIs [e.g., (Cincotti et al., 2008)]. However, calibration time may still exceed a child’s attention span, especially if the stimuli are not inherently interesting. Traditionally, P300 BCIs use a fixed duration of calibration data. Our various studies used 9, 13, or 19 min of calibration data (Alcaide-Aguirre et al., 2017; Huggins et al., 2019, 2020). While calibration data of this duration is not necessarily essential, calibrations performed on small amounts of data may report success, but not correctly interpret new data. Methods to create BCIs that work without individualized calibration or that perform more rapid calibration using transfer learning from past participants are not yet readily available [e.g., (Lu et al., 2009; Xu et al., 2015; Sahay and Brinton, 2021)]. Methods are needed to automatically remove data with artifact or low participant attention. In addition, methods are needed to rapidly validate, preferably during collection of the calibration data, whether a calibration will generalize to new data.

### Electrode location

An additional consideration for calibration is electrode location. Most P300 BCI studies use similar electrode locations (Thompson et al., 2009; Wolpaw et al., 2018) based on experiments with typically developing participants and people with ALS. For these groups, customizing electrode locations appears to actually reduce performance (Colwell et al., 2014). However, for people with congenital disabilities, custom electrode locations can be useful and perhaps vital for successful calibration (Tou et al., in revision). This is not surprising considering that neuroanatomy imaging of children with CP found abnormal results in 80–90% (Korzeniewski et al., 2008). Thus, when working with children with congenital neurodevelopmental conditions, custom electrode locations should be considered if calibration with standard locations fails. Further, ongoing efforts to reduce the number of electrodes used by BCIs should consider the possible necessity of atypical electrodes locations.

### Electroencephalogram headgear challenges

EEG headgear designed for people without impairments may not fit the heads of people with multiple congenital impairments. Prevalence of head asymmetry among people with the most severe impairments from CP are reported as over 40% (Kawakami et al., 2013) and microcephaly at 30–60% (Venkateswaran and Shevell, 2008; Minciu, 2012). We have repeatedly experienced issues with poor fitting gel and dry EEG headgear. Thus, there is a need for either custom fitted headgear or headgear that accommodates asymmetries or atypical head shapes.

Additionally, the time required for EEG electrode setup and establishing good recording quality can be tedious. Providing caregiver-suggested/provided entertainment can help. In addition, the necessary intrusion into a child’s personal space and the strange sensations of gel electrodes or the weight of a dry electrode headset may be poorly tolerated. Comfortable EEG headgear that can be rapidly set up and quickly establish good recording quality is essential. Further, it may be necessary to specifically acclimate children so that they will tolerate the headgear.

Electroencephalogram signals may also be vulnerable to movement artifact. Children, of course, move; and children with CP may have more frequent and less controlled movements, leading to greater issues with movement artifact in EEG. Further, movements may bring the EEG headgear into contact with the back of a chair or a wheelchair headrest, which can create EEG artifact, dislodge the headgear, or be painful (Daly et al., 2013).

## Discussion

**FIGURE 2:**
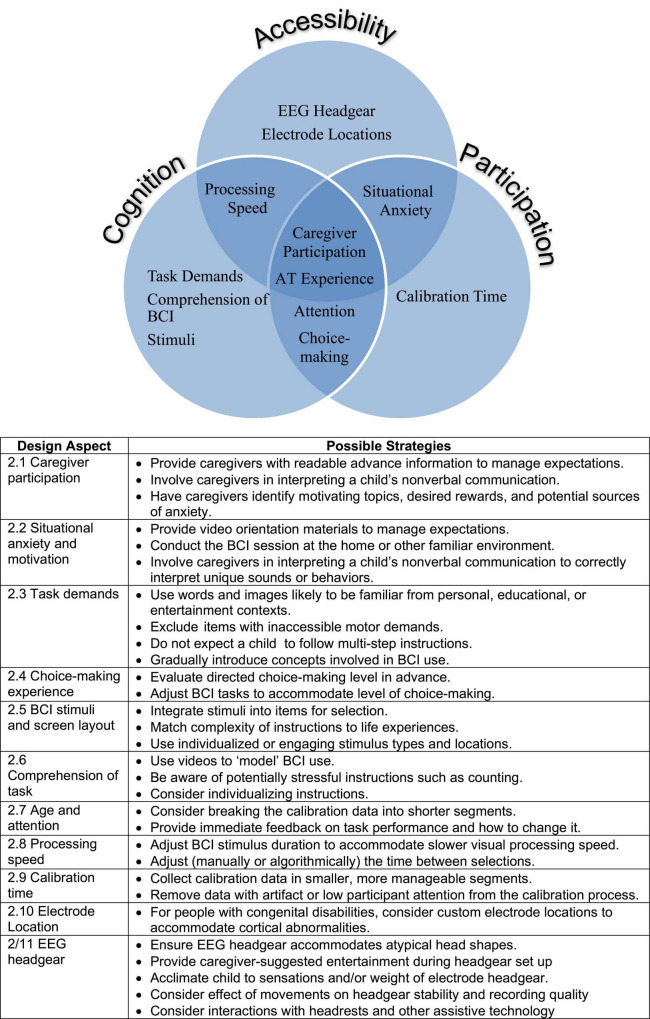
Concept map, design aspects, and possible strategies to consider in brain-computer interfaces (BCI) design for children with disabilities.

For any child, familiarity and a paced and supportive introduction of the EEG headgear and the tasks involved in BCI use may be vital for eventual success. However, for children with impairments in speech and volitional movement, including those with CP, factors related to agency, neuroanatomy, and lived experience may differ in important ways. Building on work-to-date, we present a concept map (Figure 2) of issues and strategies to consider when designing BCIs for children with multiple impairments.

Consideration should first be given to overall accessibility, not only for wheelchair access, but also for compatibility of EEG headgear with seating systems and any known neuroanatomy anomalies. Slowed visual processing speed or a history of cognitive visual impairments should be considered. Children with CP are at significant risk for epilepsy and a history of photosensitive epilepsy is usually an exclusion factor for BCI use, though we have not seen reports of visual BCI stimuli actually causing seizures. Next, the cognitive and experimental aspects of the BCI design should be considered. Successful participation may depend upon implicit and explicit assumptions about what the child knows and can understand, and their previous experience with alternative access methods such as switch or eye-gaze technology. Engagement will likely be affected by comfort and familiarity with the nature and complexity of the stimulus presentation. If participation requires following directions, how will that fundamental capability be assessed, or will it be developed during training? The child’s endurance and sustained attention will affect the time available for setup, training, and assessment. In addition to situational anxiety, travel time can affect attention and fatigue. What can be done to maximize the child’s comfort and motivation? These considerations will inform strategic planning of where (home, clinic, or lab) to conduct the assessment and over how many sessions. More sessions allow introduction of new concepts at a slower pace but also increase travel burden if not conducted in the home. The child’s engagement and participation may depend upon the extent to which the study requires conscious compliance with instructions rather than initial passive participation, which can ease the child into BCI use. Thus, accessibility, cognition, and participation considerations establish the basis on which successful BCI calibration and successful adoption of BCI as an access method will be built.

## Data availability statement

The original contributions presented in this study are included in the article/supplementary material, further inquiries can be directed to the corresponding author.

## Ethics statement

The studies involving human participants were reviewed and approved by University of Michigan Institutional Review Board IRBMED and the Cerebral Palsy Alliance Human Research Ethics Committee. Written informed consent to participate in this study was provided by the participant or the participants’ legal guardian/next of kin. Written informed consent was obtained from the individual(s) for the publication of any potentially identifiable images or data included in this article.

## Author contributions

JH and PK contributed to the analysis of data. JH and PK with their staff, performed most of the data collection for the studies, with some participation from SW. JH drafted most of this manuscript with many paragraphs contributed in their entirety by SW and PK. All authors collaborated on the design of the studies.
